# Inspiratory muscle strength and six-minute walking distance in heart failure: Prognostic utility in a 10 years follow up cohort study

**DOI:** 10.1371/journal.pone.0220638

**Published:** 2019-08-01

**Authors:** Sergio Henrique Rodolpho Ramalho, Gerson Cipriano Junior, Paulo José Cardoso Vieira, Eduardo Yoshio Nakano, Eliane R. Winkelmann, Carine C. Callegaro, Gaspar Rogério Chiappa

**Affiliations:** 1 Health Sciences and Technologies Program, University of Brasília, Brasília, Brazil; 2 Rehabilitation Sciences Program and Health Sciences and Technologies Program, University of Brasília, Brasília, Brazil; 3 Intensive Care Unit of the Hospital Cristo Redentor, Porto Alegre, Brazil; 4 Department of Statistics, University of Brasilia, Brasília, Brazil; 5 Universidade Regional do Noroeste do Estado do Rio Grande do Sul-UNIJUI, Ijui, Brazil; 6 Department of Physiotherapy, Federal University of Santa Maria, Santa Maria, Brazil; 7 Faculdades Integradas da União Educacional do Planalto Central, Brasília, Brazil; Universita degli Studi di Napoli Federico II, ITALY

## Abstract

**Background:**

Maximal inspiratory pressure (PI_max_) and 6-minutes walk distance test (6MWD) may be more available and feasible alternatives for prognostic assessment than cardiopulmonary testing. We hypothesized that the PI_max_ and 6MWD combination could improve their individual accuracy as risk predictors. We aimed to evaluate PI_max_ ability as a mortality predictor in HF and whether the combination to 6MWD could improve risk stratification.

**Methods:**

Prospective cohort from HF Clinics of three University Hospitals. PI_max_, 6MWD and pVO_2_ were obtained at baseline. The end point was all cause mortality.

**Results:**

Consecutive 256 individuals (50% woman, 57.4±10.4years) with low ejection fraction (LVEF) (31.8±8.6%) were followed up to 10years. During a median follow-up of 34.7 (IQR 37) months, 110 participants died. Mean±SD values were: pVO_2_ 14.9±5.1mL/kg/min, PI_max_ 5.5±1.3kPa and 6MWD 372±118m. In multivariate Cox regression, pVO_2_, PI_max_, 6MWD and LVEF were independent mortality predictors. The pVO_2_ showed gold standard accuracy, followed by PI_max_ (AUC = 0.84) and 6MWD (AUC = 0.74). Kaplan-Meier mean survival time (MST±SE) for lower (≤5.0kPa) and higher (>6.0kPa) PI_max_ tertiles, were 37.9±2.8months and 105.0±5.2months respectively, and addition of 6MWD did not restratified risk. For intermediate PI_max_ tertile, MST was 81.5±5.5months, but adding 6MWD, MST was lower (53.3±7.6months) if distance was ≤350m and higher (103.1±5.7months) for longer distances.

**Conclusion:**

PI_max_ is an independent mortality predictor in HF, more accurate than 6MWD and LVEF. Addition of 6MWD empowers risk stratification only for intermediate PI_max_ tertile. Although less accurate than pVO_2_, this simpler approach could be a feasible alternative as a prognostic assessment.

## Introduction

The assessment of prognostic markers in heart failure (HF) supports therapeutic decisions[1], and promotes open communication between clinicians and patients on the goals of therapy[2]. Impaired functional capacity is related to prognosis in HF in a severity-dependent manner. The peak oxygen consumption (pVO_2_) is a reference functional measure from the cardiopulmonary exercise test (CPX), able to predict the likelihood of death in HF[3, 4]. Nevertheless, CPX is not available in all healthcare settings, such as in middle to low income countries, due to relatively expensive technology and required professional expertise. In such scenarios, clinicians need to resort to alternative methods to estimate risk.

One widely adopted option to CPX is the six-minutes walk distance test (6MWD). An inexpensive test that reproduces functional capacity for submaximal activities and provides prognostic information in different etiologies of HF[5]. Yet, coexisting morbidities, cognition deficits, among other cofactors can influence reproducibility and limit extrapolation of its prognostic power[6]. Alternatively, the maximal inspiratory pressure (PI_max_) is a low-cost metric of inspiratory muscle strength—a reliable marker for estimated maximal work of breathing- and is related to disease severity in chronic HF. PI_max_ independently predicts mortality in HF[7–9] and can be obtained in individuals unable to perform an exercise test[10], despite relatively underused in clinical practice. However, it has not been determined if the combination of PI_max_ and 6MWD methods can improve risk stratification in HF compared to their individual performances or how comparable this combination is to pVO_2_.

Therefore, in a long-term HF cohort, our aims were: 1) to determine whether the combination of PI_max_ to 6MWD could improve risk stratification in HF; and 2) to define the accuracy of their combination to predict mortality, compared to pVO_2_. We hypothesized that combining PI_max_ to 6MWD improves their accuracy as mortality risk predictors.

## Methods

### Participants and design

This prospective cohort recruited a total of 256 consecutive participants referred to Heart Failure and Transplant Clinics at three University Hospitals from the State of Rio Grande do Sul-Brazil, where data was collected between January/2001 and December/2009. The local ethics committee (Hospital de Clínicas de Porto Alegre, protocol number 08–589) approved the study and all participants signed an informed consent form.

All evaluations were undertaken on outpatient basis and data were part of standard care. The inclusion criteria to be enrolled in the cohort were: ≥18years, HF from any etiology, left ventricular ejection fraction (LVEF) <50% by echocardiography. HF was diagnosed by cardiologists through clinical assessment, considering current or prior signs and symptoms of HF syndrome^1^ and a low ejection fraction at enrollment, irrespective of etiology. All participants were previously sedentary (<150 minutes of moderate physical activity/week). They should be clinically compensated and on stable pharmacologic treatment for at least 3 months previously to enrollment. We excluded patients already engaged on cardiac rehabilitation programmes, or previously diagnosed moderate to severe chronic pulmonary disease, dialytic renal failure or other severe illnesses with reduced life-expectancy (particularly acquired immunodeficiency syndrome or cancer), those unable to walk unassisted or unable to exercise because of noncardiac limitations.

At baseline, individuals underwent a cardiology consult, electrocardiogram, laboratory tests and echocardiography. Medications in use were ascertained at baseline only. Based upon their clinical condition, they returned every 3, 6 or 12 months. All patients were classified as classes C or D of the American Heart Association (AHA)[1]. The outcome of interest was overall mortality. Vital status was evaluated directly from patients or their relatives, on hospital visits, from hospital records, by telephone contact or assessing yearly a local state death certificate database.

### Six minute-walk distance test

Participants performed a 6MWD test at baseline, according to established American Thoracic Society Guidelines[11]. After resting seated for 10 minutes, they were instructed to walk as fast and as long as possible, in a 30meters obstacle-free corridor, limited by turnaround cones. Standardized verbal encouragement was given every minute. After 6 min, they were instructed to stop, and the total distance was measured, rounding to the nearest meter.

### Inspiratory muscle strength

Inspiratory muscle function test was performed using a digital pressure transducer (MVD-500 V.1.1 Microhard System, Globalmed, Porto Alegre, Brazil), connected to a system with two unidirectional valves (DHD Inspiratory Muscle Trainer, Chicago, Illinois)[12]. Maximal static inspiratory pressure (PI_max_) was determined in deep inspiration from the residual volume, against an occluded airway with a minor air leak (2 mm). The highest value of six measurements was used for analysis. Reference values considered age, gender, and weight[13].

### Cardiopulmonary exercise test (CPX)

Cardiologists conducted a maximal incremental (10W/min ramp) exercise test, performed on an electrically braked cyclergometer (ER-900, Ergoline, Jaeger, Wurzburg, Germany). Pedaling frequency was maintained at 60rpm. The test was terminated upon fatigue, cardiovascular symptoms, evidence of ischemia or arrhythmia. Before each test, the device was calibrated using reference 3L volume syringe and prespecified gases. Heart rate, minute ventilation, oxygen uptake (VO_2_, STPD), carbon dioxide production (VCO_2_, STPD) and other CPX variables were acquired breath-by-breath (Metalyzer 3B, CPX System, Cortex, Leipzig, Germany). Due to baseline oscillations and to expected oscillatory breathing in some patients, measures were averaged over 10second intervals for standardized analysis[14]. Peak VO_2_ (mL/kg/min) was defined as the highest value achieved during the test[14]. The oxygen uptake efficiency slope (OUES) was calculated as the slope of the regression between minute ventilation (log10) and VO_2_. The 10-second averaged PETCO_2_ at maximal exercise was also determined[15].

### Statistical approach

Data were reported as mean±SD or absolute numbers and percentages as applied. Cox regression was used to estimate the relationship of PI_max_ and 6MWD and overall mortality, adjusted to potential confounders to the association of interest: pVO_2_, LVEF, New York Heart Association (NYHA) classes I and II, use of angiotensin converting enzyme inhibitors (ACEi) or angiotensin receptor blocker (ARB), use of betablockers, ischaemic etiology, age, implantable devices, serum creatinine and atrial fibrillation. Such adjustments are associated to morbidity or mortality in HF or could influence the performance on the functional tests considered in this study. Assessment of the proportional hazard assumption was performed using residual plots against rank time. Receiver operating characteristic (ROC) curves were constructed to determine the accuracy of PI_max_ and 6MWD as individual measures, and as a combined model, to discriminate mortality. Hanley & McNeil test was used to compare Areas Under the Curves (AUC). Kaplan-Meier analysis were subsequently performed, from enrollment date until the last registry of follow-up or death. For this analysis, tertiles of PI_max_ were used: highest (>6.0kPa); lowest (≤5kPa); and the intermediate (>5.0 and ≤6.0kPa). This categorization was preferred for clinical applicability, accounting to potential variations in PI_max_ value from different sites and to variability of inspiratory muscle weakness criteria[16]. While for 6MWD, dichotomization considered the best cut-off value from ROC curve (≤350m or >350m). We further determined if adding the 6MWD performance to PI_max_ could improve risk stratification, compared to isolated PI_max_. Time to death is expressed as mean±SE with respective 95% confidence interval and compared by the log-rank test.

To identify potential sources of variability we performed Kaplan-Meier analysis comparing male *versus* female, diabetic *versus* non-diabetic and those with *versus* without previous stroke, for PI_max_ and 6MWD strata. Additionally, we used an unpaired t-test to compare means of the main variables between two age groups (<65 and ≥65 years). The p-value used to reject null hypothesis was <0.05. Power of Cox regression was calculated *a posteriori* (*stpower cox* STATA command). Microsoft Excel 2010, IBM-SPSS version 20 and STATA version 14.2 for Mac were used.

## Results

### Cohort characteristics

Of the 256 participants, half were women, aged 57±10years (Table 1). LVEF ranged from 10% to 49% and averaged 32%. Ischaemic etiology was the most frequent, followed by idiopathic cause (22%). No patient underwent cardiac transplantation. At baseline, most participants presented NYHA classes I or II, and >70% used ARB/ACEi and/or beta-blockers. Eighty-five patients (33.2%) achieved more than 70% of predicted PI_max_. Survivors averaged 6.1±1.1kPa and non-survivors 4.6±1.1kPa of PI_max_, 412±99m and 319±121m for 6MWD and 33±8% and 30±9% for LVEF respectively. Average pVO_2_ was 19±3mL/kg/min for survivors and 10±2mL/kg/min for non-survivors. On 6MWD, 139 patients (55.6%) reached at least 350 meters. Median follow up was 34.7 months (25-75^th^ percentile 21.9–58.9). Total of 110 patients (43%) died.

**Table 1:** Baseline overall demographic and clinical characteristics.

Age, years (mean±SD)	57.4±10.4
Male gender, n (%)	128 (50)
Height, cm (mean±SD)	164±9
Weight, kg (mean±SD)	74.3±13.2
Body Mass Index, kg/m^2^ (mean±SD)	27.5±3.3
Ischaemic etiology, n (%)	80 (35.0)
NYHA Classes I and II, n (%)	171 (83.4)
NYHA Class I, n(%)	92 (45)
NYHA Class II, n(%)	79 (39)
NYHA Class III, n(%)	29 (14)
NYHA Class IV, n(%)	5 (2)
CABG, n (%)	27 (11.6)
Stroke, n (%)	21 (9.1)
*Diabetes mellitus*, n (%)	54 (23.3)
Hypertension, n (%)	106 (45.9)
Current medications	
Betablockers, n (%)	163 (70.6)
ACEi or ARB, n (%)	173 (74.9)
Spironolactone, n (%)	59 (25.5)
Use of devices* n (%)	57 (24.0)
Implantable cardioverter-defibrillator	34 (14)
Cardiac resynchronization therapy	4 (2)
Pacemaker	19 (8)
Electrocardiogram	
Atrial fibrillation, n (%)	51 (26.6)
Left bundle branch, n (%)	63 (28.4)
Echocardiography (mean±SD)	
Left atrium diameter, cm	4.9±1.0
LV diastolic diameter, cm	6.7±0.9
LV ejection fraction, %	31.8±8.6
Laboratory tests (mean±SD)	
Creatinine, mg/dL	1.3±0.4
Urea, mg/dL	59.4±29.8
Sodium, mEq/L	140.3±3.7
PI_max_, kPa (mean±SD)	5.5±1.3
6MWD, m (mean±SD)	372.2±117.9
Peak VO_2_, mL/kg/min (mean±SD)	14.9±5.1
RQ (mean±SD)	1.04±0.11

n (%): number of patients and percent of non-missing data; SD: standard deviation; NYHA = New York Heart Association; CABG = Coronary angioplasty bypass; ACEi = Angiotensin-converting enzyme inhibitor; ARB = angiotensin receptor blocker; LV = left ventricle. PI_max_: maximal inspiratory pressure; 6MWD: 6-minute walk test distance; RQ = respiratory quotient

*pacemaker, implantable cardioverter-defibrillator or resynchronization therapy.

### Risk predictors and accuracy

Four variables remained independent in the multivariate Cox regression model: pVO_2_, PI_max_, 6MWD, LVEF (Table 2). Accounting for all covariates, pVO_2_ and PI_max_ showed each 23 and 24% lower likelihood of death per increase in measured unit (mL/kg/min and kPa, respectively), per month of observation. Residual plots showed no significant interaction between rank time and pVO_2_, PI_max_, LVEF or 6MWD (S1 Fig). Considering the hazard ratio relative to PI_max_, adjusted for the independent variables; the distribution of PI_max_; the overall mortality rate; and the sample size, the observed power was 0.94.

**Table 2:** Cox regression analysis of variables of interest for mortality outcome.

	Univariate			Multivariate		
	X^2^	HR (95% CI)	p	X^2^	HR (95% CI)	p
Peak VO_2_	87.29	0.722 (0.674–0.773)	<0.001	27.40	0.771 (0.699–0.850)	<0.001
PI_max_	79.76	0.530 (0.461–0.609)	<0.001	6.60	0.760 (0.617–0.937)	0.01
6MWD	38.32	0.995 (0.993–0.996)	<0.001	12,77	0.996 (0.993–0.998)	<0.001
LVEF	7.44	0.971 (0.950–0.992)	0.006	12.98	0.951 (0.925–0.977)	<0.001
Use of ACEi/ARB^#^	5.88	1.686 (1.105–2.573)	0.015			0.943
Use of betablockers^#^	3.14	1.461 (0.960–2.221)	0.077			0.075
Creatinine	2.17	1.330 (0.910–1.944)	0.141			0.893
NYHA Classes I and II^#^	0.95	0.759 (0.436–1.322)	0.331			0.698
Ischaemic etiology^#^	0.51	0.861 (0.572–1.297)	0.476			0.190
Atrial fibrilation^#^	0.439	1.213 (0.685–2.148)	0.508			0.984
Age	0.356	0.995 (0.978–1.012)	0.551			0.528
Use of Devices*^#^	0.012	0.973 (0.569–1.591)	0.915			0.887

HR: hazard ratio; CI: confidence interval; PI_max_: maximal inspiratory pressure; 6MWD: 6-minute walk test distance; LVEF = left ventricle ejection fraction; ACEi = Angiotensin-converting enzyme inhibitor; ARB = angiotensin receptor blocker; NYHA = New York Heart Association

*pacemaker, implantable cardioverter-defibrillator or resynchronization therapy.

# Categorical variables.

Individually, pVO_2_ showed gold-standard accuracy in discriminating mortality (S1 Table), followed by PI_max_ (AUC 0.84) and 6MWD (AUC 0.74), while LVEF was not discriminative (AUC 0.57). PI_max_ AUC was greater than 6MWD (p = 0.017). Composite models (PI_max_+6MWD; PI_max_+ LVEF; and PI_max_+6MWD+LVEF) showed slightly higher AUCs (0.88; 0.85; and 0.89, respectively), without significant differences in discriminating mortality than did PI_max_ alone (p>0.05 for paired comparisons). Most accurate cutoffs for PI_max_ was 5.4kPa (sensitivity 78% and specificity 74%) and for 6MWD, 350m (sensitivity 69% and specificity 72%). PI_max_ tertiles cutpoints were 6.0kPa (sensitivity 89% and specificity 49%) and the lower was 5.0kPa (sensitivity 64% and specificity 91%).

### Kaplan-Meier analysis

At any time-point, a higher risk of death was observed for the lower PI_max_ tertile compared to the highest tertile, particularly for the weakest stratum (S2 Fig). Survival times were almost threefold higher if PI_max_>6kPa and more than twice higher if PI_max_ was between 5 and 6kPa, than it was if PI_max_ was ≤5kPa (Table 3). Similarly, those who could not reach 350m in 6MWD were at higher risk of death then those who reached further distance (S3 Fig).

**Table 3:** Kaplan-Meier (KM) analysis estimation of mean survival times (in months) for Maximal Inspiratory Pressure and Six Minute Walk Distance Test considering the entire observation period.

		n (censors)	KM Mean±SE (months)	CI 95%
**Maximal Inspiratory Pressure**				
≤5.0 kPa		84 (13)	37.86 ± 2.82	(32.32–43.38)
>5.0 and ≤6.0 kPa		85 (58)	81.54 ± 5.55	(70.66–92.41)
> 6.0 kPa		87 (75)	105.02 ± 5.22	(94.78–115.27)
**Six Minute Walk Test Distance**				
≤ 350m		117 (41)	48.52 ± 3.97	(40.73–56.31)
> 350m		139 (105)	87.60 ± 4.49	(78.79–96.41)
**Combinations**				
	≤5.0 kPa and ≤350m	50 (2)	30.78 ± 2.96	(24.98–36.58)
	≤5.0 kPa and >350m	34 (11)	49.86 ± 5.03	(39.99–59.72)
	>5.0 and ≤6.0 kPa and ≤350m	34 (14)	53.33 ± 7.59	(38.46–68.20)
	>5.0 and ≤6.0 kPa and >350m	51 (44)	103.12 ± 5.72	(91.91–114.33)
	>6.0 kPa and ≤350m	33 (25)	90.22 ± 10.96	(68.64–111.70)
	>6.0 kPa and >350m	54 (50)	111.68 ± 5.20	(101.48–121.89)
**Overall**		**256 (146)**	**68.82** ± **3.31**	**(62.32–75.31)**

n: number of patients; SE: standard error; CI: confidence interval.

**Fig 1:**
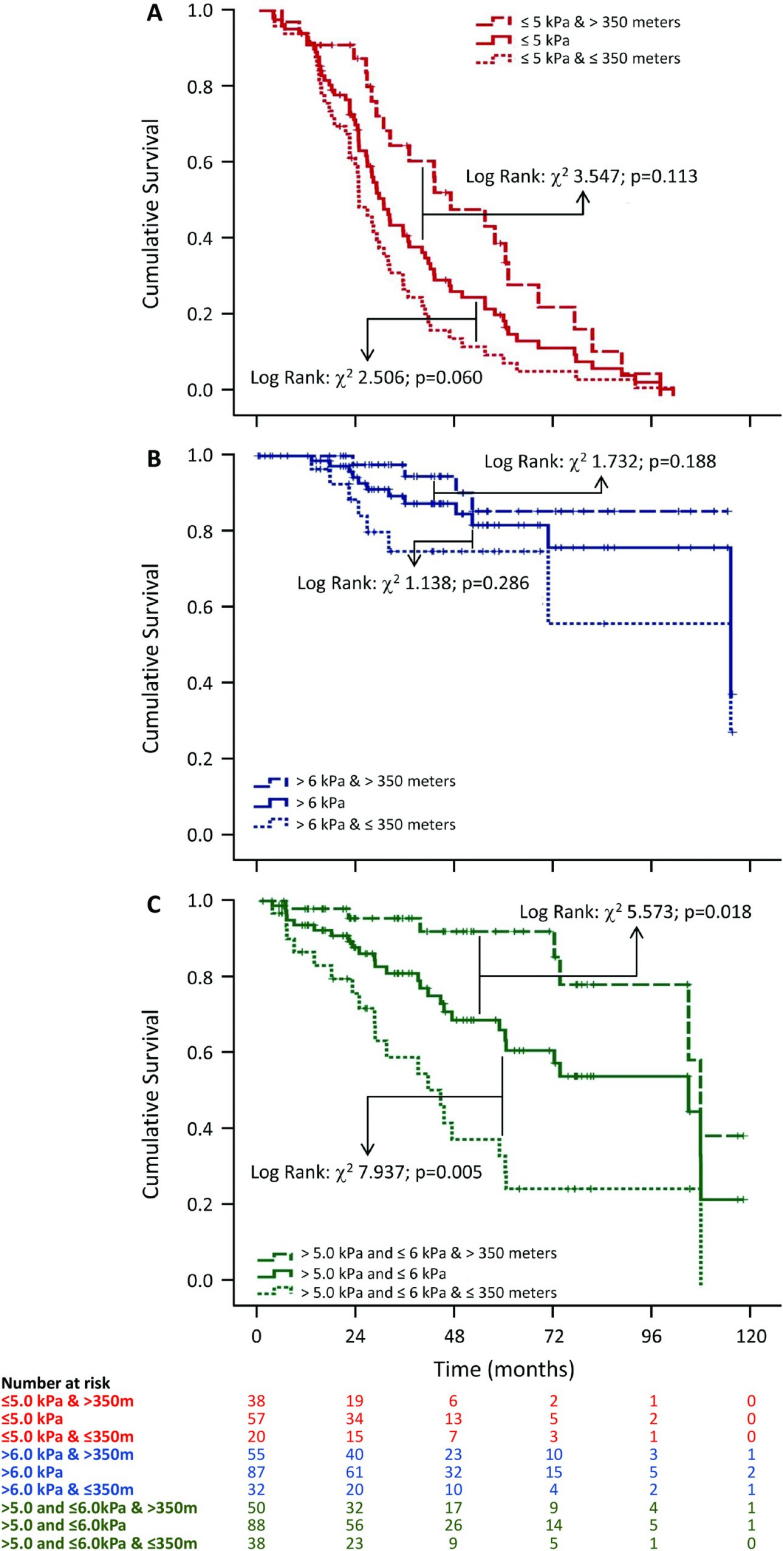
Kaplan-Meier survival curves for combined maximal inspiratory pressure and six-minutes walk distance test in low ejection fraction heart failure patients. Kaplan-Meier analysis shows no statistical differences between PI_max_ alone or combined to 6MWD, considering its lowest (A) or highest tertiles (B) in survival probability. Significant additive effect of 6MWD was observed only in intermediate PI_max_ tertile (C).

In the Lowest (Fig 1A) or in the Highest (Fig 1B) PI_max_ tertiles, no significant difference in survival times was observed when 6MWD performance was added (Table 3). Nevertheless, in the Intermediate tertile (Fig 1C), a worst performance in 6MWD significantly decreased survival time, while the opposite happened if the achieved distance was >350m.

Accounting to potential sources of variability, differences in gender, diabetes and stroke prevalences were analyzed separately. Log-rank showed non-significant differences between Kaplan-Meier comparisons of male *versus* female, diabetic *versus* non-diabetic and those with previous stroke *versus* those without, whichever PI_max_ or 6MWD stratified analysis (S2 Table). Distributions of pVO_2_, PI_max_, 6MWD, LVEF were similar between <65 and ≥65 years old groups (S3 Table), and Cox regression showed that the association of each with mortality persisted when adjusting for age group: pVO2 [HR 0.724 (95%CI 0.676–0.775); p<0.001], PI_max_ [HR 0.533; (0.463–0.613); p<0.001], 6MWT [HR 0.995 (0.993–0.996); p<0.001], LVEF [HR 0.966 (0.945–0.987); p = 0.002].

## Discussion

The main findings of this prospective long-term cohort study with 256 HF patients reinforces that the severity of inspiratory muscle weakness (IMW), measured by PI_max_, and shorter walking distance, by 6MWD, proportionally increases mortality risk, but this outcome is more accurately discriminated by the PI_max_. Notably, only in patients within the intermediate PI_max_ tertile (>5.0 and ≤6.0kPa), the combination of 6MWD performance significantly altered mean survival time, improving it if distance was >350m and decreasing it if distance was ≤350m, when compared to the isolated PI_max_ effect. Within other tertiles, mortality risk from PI_max_ remained independent of knowing walked distance.

Prognostic value of PI_max_ has been studied before [7–9, 17]. In the present study, we confirmed that the lower PI_max_, the higher mortality in HF with reduced ejection fraction, discriminating risk more precisely than 6MWD or LVEF, but as expected, less accurately than pVO_2_. Discriminatory risk accuracy by AUC (S1 Table), quantifies how separated is the distribution of means between survivors and non-survivors. Indeed, this was demonstrated as distribution of pVO_2_ from survivors are mostly separated from non-survivors (9mL/kg/min difference between means, representing 47% variation), followed by PI_max_ (1.5kPa difference, 25% variation) and 6MWD (93m difference, 23% variation), all with significant AUC ≥0.74; while LVEF had an overlapped distribution among survivors and non-survivors (3 percentage points difference, 9% variation), which poorly discriminated groups (AUC 0.57, p = 0.07). In contrast to Myers et al[7], PI_max_ AUC for mortality was higher in our study, where the majority of patients (65%) achieved a PI_max_ up to 6.0kPa, and 67% was considered to have IMW[13], showing lower inspiratory strength on average comparing to other studies[7, 8]. This reinforces that, even in more severe IMW, PI_max_ absolute values independently represent a strong prognostic marker in HF.

Considering possible variations in PI_max_ absolute values and variable IMW diagnostic criteria, we also extended this analysis in tertiles, for a practical clinical approach. PI_max_ remained an independent factor in the lowest tertile—with the greatest death risk—and in the highest tertile, with the best prognosis. However, those in the intermediate PI_max_ tertile could be restratified according to 6MWD performance.

The muscle hypothesis[18], a generalized syndrome of muscle dysfunction in chronic HF, might be the main rationale for the cardiopulmonary and skeletal muscle function relationship, and the associated mortality risk in HF. A vicious cycle of inflammation, oxidative stress, and hypoperfusion generates more muscle atrophy and dysfunction, worsening HF[18]. Thus, some functional measurement overlap is expected according to the “muscle hypothesis” concept in HF for PI_max_, maximal (pVO_2_) and submaximal (6MWD) exercise capacity metrics[7], which may impact unequally PI_max_ subgroups.

Reduced pVO_2_ in HF represents a global impairment in cardiac, pulmonary and peripheral muscle systems, which reflects disease severity and is considered a universal prognostic marker[4]. Furthermore, impaired inspiratory power proportionally reduces ventilatory response to stress, precludes adequate pulmonary and peripheral gas exchange, and impairs efficient biochemical metabolites washout[10, 19]. Clinically, IMW is associated with dyspnoea, poor exercise tolerance and reduced functional status in patients with HF[10, 19]. While pVO_2_ is a more robust HF severity marker, PI_max_ can be considered a global “work of breathing” metric in HF, beyond an isolated inspiratory muscle measure[7], and can represent a reasonable alternative to pVO_2_ for mortality risk stratification, as demonstrated.

Notably, alterations in structure and function of inspiratory muscles seem more pronounced than in other skeletal muscles with progressive HF[7, 19]. Diaphragm—the main inspiratory muscle—has extrinsic automaticity and is under constant workload, increased in HF, differently from limb muscles which alternate activity/rest cycles[20]. Indeed, chronic adaptation in diaphragm of HF patients differs from limb muscles, where a shift from fast to slow myosin heavy chain isoforms is observed, with an increase in oxidative capacity and a decrease in glycolytic capacity, as a result of increased work of breathing[21]. This particularity is consistent to the independent PI_max_ performance as a mortality predictor among other functional variables, in our analysis and in others[7, 9]. Additionally, it could partially explain the influence of 6MWD on PI_max_. In the intermediate PI_max_ tertile, damage to inspiratory muscle fibres may be partial and heterogeneously distributed, and addition of the submaximal effort capacity (6MWD) provided significant prognostic information. While in the extremes PI_max_ tertiles, healthier or severely damaged inspiratory muscle function outperformed 6MWD prognostic value.

### Clinical perspective

IMW is prevalent in HF, present in 30–50% as outpatients[12] and approximately in 70% of elderly patients admitted with acute HF[22]. Routine screening for IMW is recommended in HF [19, 23, 24], however an arbitrary assumption of IMW as <70% of predicted PI_max_[12] may have acceptable sensitivity but lacks specificity as a prognostic parameter. Alternatively, stratification in high, intermediate and low PI_max_ may help clinicians to estimate HF mortality risk and the necessity of further testing. Inspiratory muscle strength can be measured in an office visit with a handheld device, independently of individual ability to exercise, with high reproducibility. Undoubtedly, key variables from CPX are the most powerful HF prognostic markers. Nevertheless, patients from low income countries or those unable access such technology may be precluded from a more accurate risk assessment and, consequently, from therapeutic adjustments. The 6MWD is a practical and widely used evaluation in HF as an alternative for prognostic assessment, superior to LVEF[25], however, different cohort characteristics, such as age, gender, comorbidities, disabilities lead to different outcome associated cutoffs[6], limiting the generalizability when used alone. Hence, PI_max_ is a functional prognostic assessment resource, more affordable than pVO_2_, easily obtained, and may help to select those who need further testing for risk stratification.

Absolute value of PI_max_ has been consistently demonstrated not only as an outcome marker, but also as modifiable risk factor. Although underused, inspiratory muscle training has benefits on exercise capacity, inspiratory muscle strength and dyspnoea[24], particularly in patients who cannot engage in conventional exercise training programs or who are severely deconditioned[26]. Our findings may also be extrapolated to older HF patients, when frailty and disability are more prevalent. We demonstrated that distribution of the main variables was similar to younger patients and the age group did not modify their association with mortality risk; however this analysis could have been underpowered to detect between-group differences. We can speculate that in such patients and in those more severely symptomatic, isolate inspiratory muscle training or combined to other methods, such as electromyostimulation[27, 28], can provide additional benefits to standard care.

### Study limitations

This study has several limitations. First, only 17% of the patients were in NYHA III/IV, probably because of enrollment criteria, however all patients were in AHA stages C or D, and NYHA class did not show association to mortality on Cox regression. Second, potential confounders or mediators were unavailable or not registered at baseline, such as nutritional status, peripheral artery disease, left ventricle diastolic function indices; as well as follow up exposures, such as pharmacologic adjustments, lifestyle changes or surgery. Third, from the CPX, only 67% of patients reached RQ≥1, however, lower RQ seems not to significantly reduce the prognostic power of peakVO_2_[29]; additionally, since our interest was on the relationship of PI_max_ and peakVO_2_, ventilatory threshold was not registered. Fourth, plasma cardiac biomarkers associated to HF mortality and to other functional variables[30] were unavailable to investigate their prognostic equivalence to PI_max_. Fifth, patients were centrally treated for HF, but came from multiple origins in surrounding communities, so, possible misinformation on other outcomes, such as causes of hospitalizations and specific causes of death, was anticipated. Thus, we opted to consider only all-cause mortality for this analysis. Minor missing clinical data persisted after searching in hospital medical records done on paper.

Potential clinical, pharmacological and non-negligible social factors as well, could have influenced underuse of the best evidence therapy with prognosis impact. Although ACEi/ARB, betablockers and spironolactone use at baseline may seem sub-optimal, it is in agreement with larger real-world cohorts[31, 32], showing lower adherence, which supports the generalizability of our findings. Hypotension, renal dysfunction, electrolyte disturbances[33] as well as social issues (accessibility and affordability) may also have contributed, especially with more severe and advanced disease patients in our study. Spironolactone was often introduced after ACEi/ARB or betablockers and seems to be more sensitive to all such factors, however it is uncertain if increasing its use could influence PI_max_ prognostic power.

### Conclusion

Our findings embody the evidence that the PI_max_ is superior to 6MWD or LVEF in HF patients in predicting long term mortality. Its performance is not so robust as pVO_2_ but showed reasonable comparability. Additionally, only when PI_max_ exhibits intermediate values (> 5.0 and ≤ 6.0kPa), combination to 6MWD empowers risk stratification. Although unduly emphasized, evaluation of inspiratory muscle strength is a valuable prognostic parameter and, potentially, a modifiable risk factor in HF patients.

## Supporting information

S1 Fig Partial residuals for main variables of interest. (PDF)

S2 Fig Kaplan-Meier survival curves for maximal inspiratory pressure tertiles in patients with low ejection fraction heart failure. (PDF)

S3 Fig Kaplan-Meier survival curves for six-minutes walk distance test strata in patients with low ejection fraction heart failure. (PDF)

S1 Table Area under ROC curve analysis for mortality prediction accuracy (110 deaths) of isolated and combined variables, and their comparisons to PI_max_ performance. (PDF)

S2 Table Kaplan-Meier comparison of confounding factors (gender, diabetes and stroke survivors) for PI_max_ and 6MWD strata. (PDF)

S3 Table Comparison of the main variables between HF patients younger than 65 years with those 65 years and older. (PDF)
