# Supplementary material for: Inspiratory muscle strength and six-minute walking distance in heart failure: Prognostic utility in a 10 years follow up cohort study
Source: PLoS One. 2019 Aug 1;14(8):e0220638. doi: 10.1371/journal.pone.0220638 (PMC6675323; doi:10.1371/journal.pone.0220638)

S1 Fig: Partial residuals for main variables of interest

Legend: A) PImax: maximal inspiratory pressure; B) 6MWD: six minutes walk distance test; C) LEVF: left ventricle ejection fraction; D) Peak VO2: peak oxygen uptake.

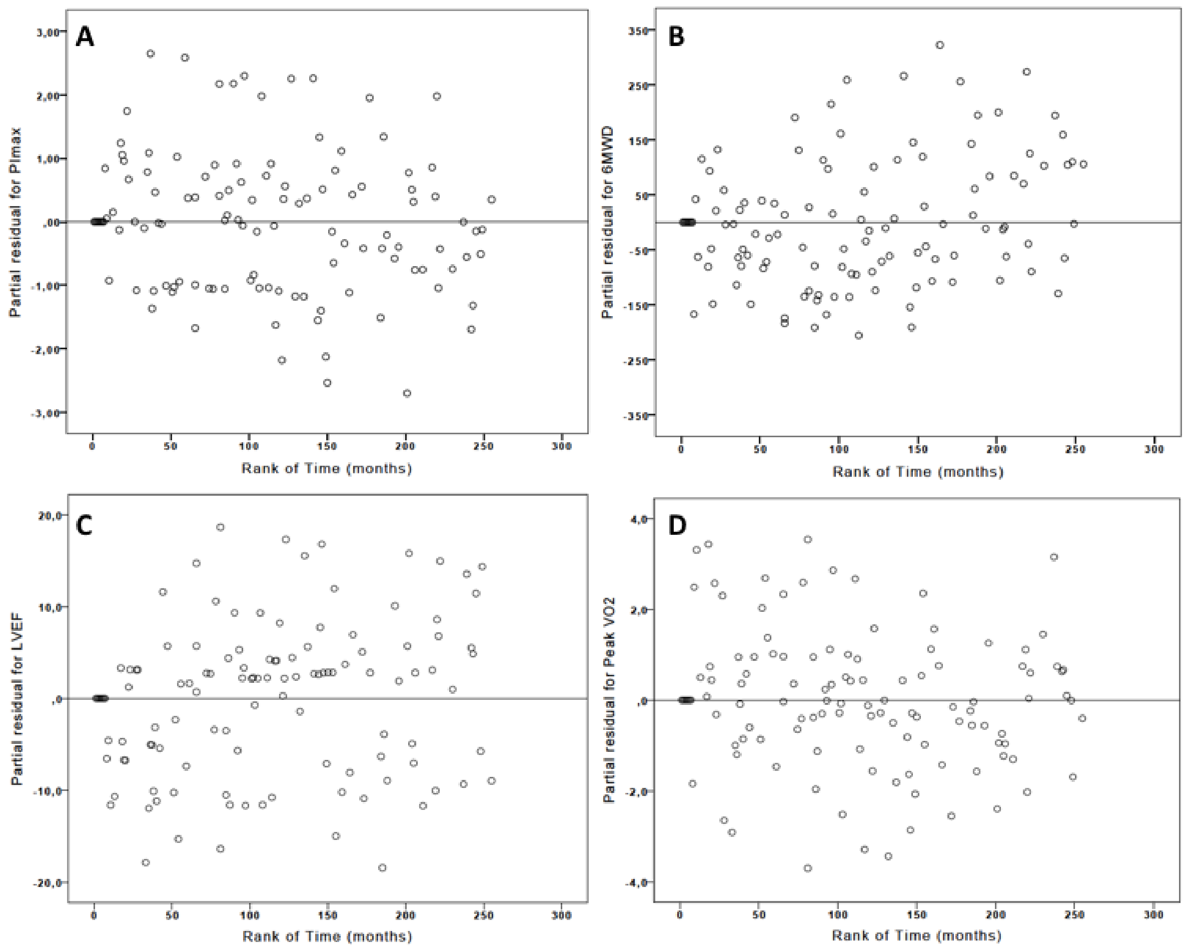

Supplement: S1 Fig — (PDF) [file pone.0220638.s001.pdf]
