# Supplementary material for: Inspiratory muscle strength and six-minute walking distance in heart failure: Prognostic utility in a 10 years follow up cohort study
Source: PLoS One. 2019 Aug 1;14(8):e0220638. doi: 10.1371/journal.pone.0220638 (PMC6675323; doi:10.1371/journal.pone.0220638)
Supplement: S2 Fig — (PDF) [file pone.0220638.s002.pdf]

S2 Fig: Kaplan-Meier survival curves for maximal inspiratory pressure tertiles in patients with low ejection fraction heart failure.

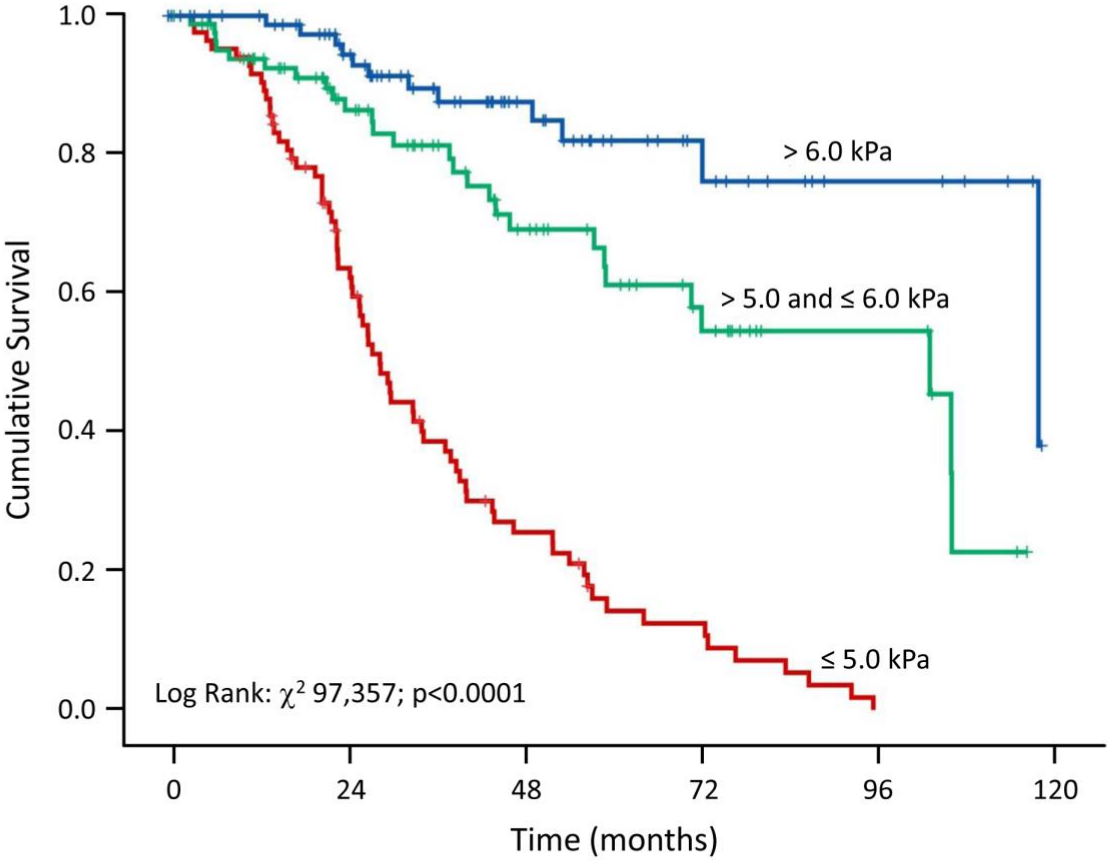

**Number at risk**						
**>6.0 kPa**	87	61	32	15	5	2
**>5.0 and ≤ 6.0 kPa**	88	56	26	14	5	1
**≤5.0 kPa**	57	34	13	5	2	0

CAPTION: Kaplan-Meier analysis shows significant differences in mortality probability between Maximal Inspiratory Pressure tertiles, during entire follow up.
