# Supplementary material for: Inspiratory muscle strength and six-minute walking distance in heart failure: Prognostic utility in a 10 years follow up cohort study
Source: PLoS One. 2019 Aug 1;14(8):e0220638. doi: 10.1371/journal.pone.0220638 (PMC6675323; doi:10.1371/journal.pone.0220638)
Supplement: S3 Fig — (PDF) [file pone.0220638.s003.pdf]

S3 Fig: Kaplan-Meier survival curves for six-minutes walk distance test strata in patients with low ejection fraction heart failure.

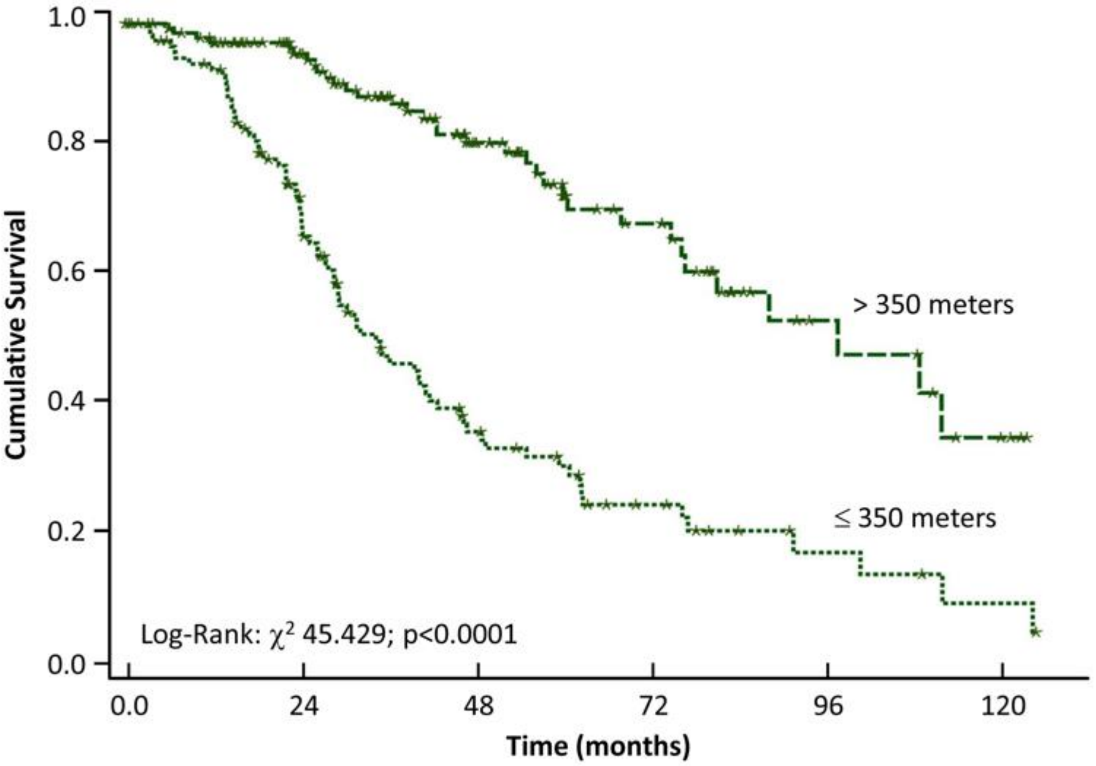

Number at risk

**>350 m**	124	87	47	23	8	1
**≤ 350 m**	108	63	25	11	4	1

CAPTION: Kaplan-Meier analysis shows significant difference in mortality probability between six-minutes walk distance strata, during entire follow up.
