# Supplementary material for: Inspiratory muscle strength and six-minute walking distance in heart failure: Prognostic utility in a 10 years follow up cohort study
Source: PLoS One. 2019 Aug 1;14(8):e0220638. doi: 10.1371/journal.pone.0220638 (PMC6675323; doi:10.1371/journal.pone.0220638)
Supplement: S1 Table — (PDF) [file pone.0220638.s004.pdf]

**S1 Table: Area under ROC curve analysis for mortality prediction accuracy (110 deaths) of isolated and combined variables, and their comparisons to $PI_{max}$ performance.**

| | AUC (±SE) | p | z-test *versus* $PI_{max}$ | p |
|---|---|---|---|---|
| Peak $VO_2$ | 1.00 ± 0.00 | <0.0001 | 6.141 | <0.0001 |
| $PI_{max}$ | 0.84 ± 0.03 | <0.0001 | - | - |
| 6MWD | 0.74 ± 0.03 | <0.0001 | 2.379 | 0.017 |
| LVEF | 0.57 ± 0.04 | 0.07 | 5.979 | <0.0001 |
| $PI_{max}$ +6MWD | 0.88 ± 0.02 | <0.0001 | -1.222 | 0.221 |
| $PI_{max}$ + LVEF | 0.85 ± 0.02 | <0.0001 | -0.298 | 0.765 |
| $PI_{max}$ + 6MWD + LVEF | 0.89 ± 0.02 | <0.0001 | -1.438 | 0.150 |

AUC: Area under the ROC curve; SE: standard error; $PI_{max}$: maximal inspiratory pressure; 6MWD: 6-minute walk test distance; LVEF = left ventricle ejection fraction.
