# Supplementary material for: Inspiratory muscle strength and six-minute walking distance in heart failure: Prognostic utility in a 10 years follow up cohort study
Source: PLoS One. 2019 Aug 1;14(8):e0220638. doi: 10.1371/journal.pone.0220638 (PMC6675323; doi:10.1371/journal.pone.0220638)
Supplement: S2 Table — (PDF) [file pone.0220638.s005.pdf]

S2 Table: Kaplan-Meier comparison of confounding factors (gender, diabetes and stroke survivors) for PImax and 6MWD strata.

Log rank (Mantel-cox)	Male *vs*. Female		With *vs* Without DM		With *vs* Without Stroke	
	X^2	p-value	X^2	p-value	X^2	p-value
**PI_{max}**						
≤5kPa	0.298	0.585	0.022	0,882	2.574	0.109
>5.0 and ≤6.0kPa	0.616	0.432	0.637	0,425	3.612	0.057
>6.0kPa	0.008	0.927	1.328	0.249	1.693	0.193
**6MWD**						
≤350m	0.005	0.941	1.171	0.279	0.750	0.386
>350m	0.197	0.657	0.523	0.469	0.672	0.412

Legend: PImax: maximal inspiratory pressure; 6MWD: six-minutes walk distance.
