# Supplementary material for: Inspiratory muscle strength and six-minute walking distance in heart failure: Prognostic utility in a 10 years follow up cohort study
Source: PLoS One. 2019 Aug 1;14(8):e0220638. doi: 10.1371/journal.pone.0220638 (PMC6675323; doi:10.1371/journal.pone.0220638)
Supplement: S3 Table — (PDF) [file pone.0220638.s006.pdf]

S3 Table: Comparison of the main variables between HF patients younger than 65 years with those 65 years and older.

**Variables**	**Adult (<65)** n=198 (77%)	**Elderly (≥65)** n=58 (23%)	**p**
Age (years), mean±SD	53.5±8.2	70.1±3.7	<0.001
Peak VO2 (mL/kg/min), mean±SD	14.7±5.1	15.7±5.2	0.174
Maximal Inspiratory pressure (kPa), mean±SD	5.4±1.4	5.6±1.3	0.578
6-munutes walk distance test (m), mean±SD	372.8±121.8	370.0±104.5	0.876
Left Ventricular Ejection Fraction (%), mean±SD	32.2±8.8	30.3±7.4	0.130
